# Chemokine Profiles and Immunosuppressive Properties of Murine Placental Nucleated Erythroid Cells in Mid- and Late Gestation

**DOI:** 10.3390/cells15161430

**Published:** 2026-08-08

**Authors:** Julia A. Shevchenko, Kirill V. Nazarov, Alina A. Gizbrekht, Tatyana A. Savostyanova, Alena P. Zakhareva, Sergey V. Sennikov

**Affiliations:** 1 Laboratory of Molecular Immunology, Federal State Budgetary Scientific Institution “Research Institute of Fundamental and Clinical Immunology” (RIFCI), 630099 Novosibirsk, Russia; shevchenkoja2023@yandex.ru (J.A.S.); kirill.lacrimator@mail.ru (K.V.N.); gizbrekht.aa17@gmail.com (A.A.G.); a.zakhareva@g.nsu.ru (A.P.Z.); 2 Institute of Medicine and Medical Technologies, Department of Immunology, Novosibirsk State University, 630090 Novosibirsk, Russia; 3 Department of Natural Sciences, Novosibirsk State University, 630090 Novosibirsk, Russia

**Keywords:** pregnancy, placenta, nucleated erythroid cells, chemokines, CCL17, CXCL9, immunosuppression, PD-L1, TGF-β, ROS, CCR3, CXCR4

## Abstract

**Highlights:**

**What are the main findings?**

- Placental CD45^+^ nucleated erythroid cells (NECs) activate three synergistic immunosuppressive mechanisms (PD-L1, TGF-β, and ROS) by late gestation, forming a redundant and robust protective system at the maternal–fetal interface.
- As pregnancy progresses, a dynamic organ-specific switching of chemokine production occurs: CCL17 and CXCL9 shift from being predominantly produced by splenic NECs at mid-gestation (E12.5) to placental NECs at late gestation (E19.5), whereas CCL22 remains constitutively high in the spleen and minimal in the placenta.

**What are the implications of the main findings?**

- Despite dynamic chemokine profile remodelling, the stable immunosuppressive capacity of placental NECs from mid-gestation onward suggests that chemokine regulation and suppressive function are independently controlled, ensuring continuous fetal protection even as local chemoattractive signals adapt to changing gestational needs.
- Enhanced immune cell migration upon CCL2/CCL4 blockade points to the involvement of atypical chemokine decoy receptors (e.g., ACKR2) in the placenta, highlighting a complex chemokine network that must be considered when targeting these pathways to prevent pregnancy complications such as preterm birth or preeclampsia.

**Abstract:**

Nucleated erythroid cells (NECs) are emerging as important immunoregulators at the feto–maternal interface, yet their chemokine profiles and functional dynamics across pregnancy remain poorly understood. Using a murine allogeneic pregnancy model (CBA × C57Bl/6), we isolated placental and splenic TER-119^+^ NECs at mid- (E12.5) and late (E19.5) gestation. Chemokine production (13-plex), chemokine receptor expression (qPCR), immunosuppressive molecules (PD-L1, TGF-β, and ROS), T-cell proliferation (CFSE), and immune cell migration (Transwell) were assessed. CD45^+^ placental NECs were the main producers of PD-L1, TGF-β, and ROS, with maximal expression at E19.5, suggesting their potential contribution to the immunosuppressive functions observed in the total TER-119^+^ population. Chemokine production showed a striking shift in CCL17 and CXCL9 from the spleen to the placenta as pregnancy advanced (E12.5 → E19.5). Splenic NECs displayed dominant expressions of CCR3 and CXCR4. Unexpectedly, CCL2 and CCL4 blockade enhanced immune cell migration toward placental NECs at E19.5. Placental nucleated erythroid cells potently suppressed T-cell proliferation at E12.5, and this suppressive capacity remained stable until full term. Placental NECs undergo dynamic chemokine reprogramming while maintaining stable T-cell suppression. The paradoxical enhancement of migration after CCL2/CCL4 blockade suggests a complex chemokine network warranting further investigation. These findings provide new insights into the immunobiology of pregnancy and may have implications for understanding pregnancy complications.

## 1. Introduction

The immunological paradox of pregnancy was formulated by Peter Medawar in 1953 [1] and remains a central question in reproductive immunology. A genetically foreign fetus, expressing paternal antigens, is not rejected by the maternal immune system, while the mother’s body maintains the ability to support a certain level of protection against infections [2]. Traditionally, fetal protection has been attributed to its isolation from the maternal immune system (the hematoplacental barrier), the expression of non-classical HLA-G molecules (in humans), and the predominance of a Th2-type immune response [3,4,5]. A successful pregnancy requires the formation of a specialized microenvironment through the presence and active interaction of various immunoregulatory cells, not passive fetal isolation [6].

Regulatory T-cells (Tregs) are the principal players in this process, actively suppressing effector responses and promoting the establishment of a tolerogenic microenvironment at the feto–maternal interface [7]. Alongside Tregs, tolerogenic decidual natural killer (dNK) cells [8], myeloid-derived suppressor cells (MDSCs) [9], and regulatory B cells [10] play important roles, collectively forming an immunosuppressive niche. The dynamics of these cell populations are tightly regulated over time, creating an “immune clock of pregnancy”, the disruption of which is associated with complications such as preterm birth and pregnancy loss [11].

Over the past two decades, it has become evident that, alongside classical immunocompetent cell subsets, nucleated erythroid cells—a non-lymphoid population whose functions have shifted significantly from oxygen transport to pronounced immunoregulatory properties—play a role in maintaining feto–maternal tolerance [12,13,14]. The demonstrated immunosuppressive role of erythroid cells in neonates [15] also points to their involvement in embryonic development. Indeed, CD71^+^ erythroid cells have been shown to mediate homeostatic immunosuppressive/immunoregulatory responses during pregnancy [16]. Furthermore, a recent multi-omic study demonstrated that erythroid cells constitute a major immunoregulatory population in murine placentas—on average, 80% and 40% of mononuclear cells at E12.5 and E19.5, respectively [17], indicating that their functional characterization is relevant for understanding the mechanisms of feto–maternal tolerance.

A key manifestation of erythroid cell immunoregulatory activity is their chemokine-mediated regulation. Chemokines regulate cell migration and communication during pregnancy, with functions extending beyond classical immunological roles. Physiological chemokine levels contribute to feto–maternal tolerance through spatiotemporal regulation, whereas their dysregulation is associated with pregnancy complications such as preeclampsia and preterm birth [18]. Despite accumulating evidence on the suppressive function of erythroid cells during pregnancy, their chemokine profile, migratory activity, and the dynamics of their functional properties throughout gestation remain incompletely understood.

Therefore, this study aimed to characterize murine placental nucleated erythroid cells in the context of allogeneic pregnancy (mid- and late gestation, E12.5 and E19.5), compared with erythroid cells from the spleens of non-pregnant mice, by evaluating their chemokine profile, receptor repertoire, ability to attract immunocompetent cells, and capacity to suppress their proliferation.

## 2. Materials and Methods

### 2.1. Isolation of Erythroid Cells from Mouse Placentas and Spleens

CBA females and C57Black/6 males were paired to ensure allogeneic pregnancy. Animals were obtained from the Laboratory Animal Breeding Facility of the Federal State Budgetary Institution “Research Institute of Molecular Medicine” at the age of 2 months. Animals were paired in individual cages with unlimited access to water and food. For this study, placentas corresponding to the second trimester (embryonic day 12.5, hereinafter E12.5) and the third trimester (embryonic day 19.5, hereinafter E19.5), as well as spleens, were collected. Spleens from non-pregnant CBA females were used as controls. For euthanasia, isoflurane anesthesia (isoflurane, “Aerran”, BAXTER HEALTHCARE Corp., Round Lake, IL, USA) was administered using an anesthesia machine for small laboratory animals (R540 Mice&Rat Animal Anesthesia Machine, RWD, Shenzhen, China), followed by cervical dislocation. Mouse placentas were minced with sharp scissors and then homogenized in a glass Potter homogenizer. The number of placentas collected per mouse ranged from 3 to 6, depending on the individual litter size. The resulting cell mass was passed through three sterile metal mesh sieves of decreasing diameter to remove large tissue fragments. Final removal of cell aggregates was performed using a 40 μm nylon mesh filter (Jet Biofil, Guangzhou, China, CSS013040). The spleen was perforated with a sterile syringe needle and washed repeatedly with the RPMI-1640 medium containing L-glutamine (Biolot, Saint-Petersburg, Russia, 1.3.4.7.). Cell suspensions were collected into 15 mL tubes (Jet Biofil, Guangzhou, China, CFT411150) and centrifuged for 10 min at 400× *g* (CM-6MT centrifuge, Elmi, Riga, Latvia).

The obtained spleen cell suspension in incomplete medium was layered onto a Ficoll–Urografin mixture with a density of 1.119 g/cm^3^, prepared from Ficoll 400 (PanEco, Moscow, Russia, X062) and sodium amidotrizoate (Trazograph^®^; UNIQUE PHARMACEUTICAL Laboratories, Mumbai, India) to create a density gradient at a Ficoll–cell ratio of 1:3, followed by centrifugation for 25 min at 400× *g* (CM-6MT centrifuge, Elmi, Riga, Latvia). After centrifugation, cells from the interphase ring were collected and washed twice in Dulbecco’s solution (Biolot, Saint-Petersburg, Russia, 1.2.4.5.). The pellet obtained after the second wash was resuspended in 1 mL of Versene solution (Biolot, Saint-Petersburg, Russia, 1.2.3.2.) supplemented with 0.5% bovine serum albumin (Biolot, Saint-Petersburg, Russia, 1.4.1.5.), and the number of splenic mononuclear cells was counted using a Celltac α hematology analyzer (Nihon Kohden, Tokyo, Japan).

### 2.2. Positive Magnetic Sorting

The obtained cell suspensions from mouse placentas and spleens were used to isolate the population of nucleated erythroid cells. For this purpose, the positive magnetic sorting method was applied using the murine marker TER-119^+^. Isolated cells were resuspended in a small volume (1 mL) of Versene solution (Biolot, Saint-Petersburg, Russia, 1.2.3.2.) supplemented with 0.5% bovine serum albumin (Biolot, Saint-Petersburg, Russia, 1.4.1.5.), to which biotinylated antibodies against the TER-119 antigen (Biolegend, San Diego, CA, USA, 116204) were added at 1 μL per 1 million cells. The suspension was incubated for 15 min at 4 °C, after which 1 mL of Versene solution (Biolot, Saint-Petersburg, Russia, 1.2.3.2.) with 0.5% bovine serum albumin (Biolot, Saint-Petersburg, Russia, 1.4.1.5.) was added, and the mixture was centrifuged for 5 min at 400× *g*. The supernatant was removed, and magnetic beads coated with streptavidin (Biolegend, San Diego, CA, USA, 480016) were added to the cell pellet at 1 μL per 1 million cells, followed by further incubation for 15 min on ice. After incubation with the beads, 2 mL of Versene solution (Biolot, Saint-Petersburg, Russia, 1.2.3.2.) with 0.5% bovine serum albumin (Biolot, Saint-Petersburg, Russia, 1.4.1.5.) was added, and the mixture was placed in a magnet for 7–8 min. Thereafter, without removing the tubes from the magnet (Biolegend, San Diego, CA, USA, 480019), the liquid was decanted into a new tube (negative fraction). TER-119^+^ nucleated erythroid cells remained attached to the tube walls inside the magnet.

The incubation step in the magnetic field was repeated once more to improve sorting purity. The collected TER-119^+^ cell fraction was centrifuged for 10 min at 400× *g*, the supernatant was discarded, and complete RPMI-1640 medium supplemented with 10% FBS (Technozerg, Moscow, Russia), 10 mM HEPES (Biolot, 1.2.6.1, Saint-Petersburg, Russia), 5 × 10^−4^ M 2-mercaptoethanol (Sigma-Aldrich, M6250, St. Louis, MO, USA), 80 μg/mL gentamicin (PanEco, A011p, Moscow, Russia), and 120 μg/mL benzylpenicillin (Sintez, Kurgan, Russia) (hereinafter referred to as complete medium) was added to the cell pellet, followed by cell counting. The TER-119^+^ population comprised >90% of the sorted cells as assessed by flow cytometry. The TER-119^+^ erythroid cell population was used for RNA isolation, flow cytometric analysis, cytokine production assays, and migration and proliferation assays. The TER-119^−^ cell population was used for RNA isolation and as splenocytes in migration and proliferation assays.

### 2.3. Phenotypic Analysis of Erythroid Cells

The phenotype of splenic erythroid cells was assessed using TER-119^+^ cells obtained by magnetic sorting. The phenotype of placental erythroid cells was assessed in the total placental cell fraction. A panel of monoclonal antibodies was used for phenotypic analysis, including CD45 Pacific Blue (Biolegend, San Diego, CA, USA, 157212), TER-119 FITC (Biolegend, San Diego, CA, USA, 116206), PD-L1 PE-Cy7 (Biolegend, San Diego, CA, USA, 124314), and TGF-β-PE (Biolegend, San Diego, CA, USA, 141306). To assess reactive oxygen species production, the dye 6-Carboxy-H_2_DCFDA (6-carboxy-2′,7′-dichlorodihydrofluorescein diacetate) (Lumiprobe, 3290-25 mg, Moscow, Russia) was used with CD45-APC (Biolegend, San Diego, CA, USA, 103112), CD71 BV 421 (Biolegend, San Diego, CA, USA, 113813), and PD-L1 PE-Cy7 (Biolegend, San Diego, CA, USA, 124314). Phenotypic analysis was performed using an Attune NXT flow cytometer (ThermoFisher, Waltham, MA, USA).

### 2.4. Macrophage Generation

Macrophages were generated for RNA isolation and assessment of chemokine receptor gene expression. To this end, red bone marrow cells were obtained from the femur of a CBA mouse (female) by threefold washing of the bone marrow canal with phosphate-buffered saline. The resulting suspension was centrifuged at 1500 rpm for 10 min to pellet the cells. The supernatant was removed, and the cells were counted in 1 mL of the complete medium. Cells were incubated in a 75-cm^2^ culture flask (TPP, 90075, Trasadingen, Switzerland) in the complete medium at a concentration of 1–1.5 million/mL with the addition of 20 ng/mL M-CSF (Biolegend, San Diego, CA, USA, 576404) in a CO_2_ incubator (HERA Cell vios 160i, ThermoFisher, Waltham, MA, USA) at 37 °C for 7 days, with medium replacement on days 4–5 of culture [19]. For use in experiments, cells were detached from the flask surface using a mixture of trypsin (Biolot, Saint-Petersburg, Russia, 1.2.2.5) and Versene solutions at a ratio of 1:3.

### 2.5. Labelling of Immunocompetent Cells with CFSE

To track migration and assess proliferative activity, immunocompetent cells (splenocytes) were stained with the vital dye CFSE (CFDA SE, Lumiprobe, 26231, Moscow, Russia). Cells (up to 5 million) were resuspended in magnetic sorting buffer at room temperature, with buffer added to adjust the suspension to a concentration of 1 × 10^6^ cells/mL. To the suspension, 2 μL of a 5 mM stock solution of CFSE was added per 1 mL of cell suspension to achieve a final working concentration of 10 μM, after which the cells were placed in a CO_2_ incubator for 10 min. To stop the staining process, a 5-fold volume of ice-cold culture medium was added to the cells, and the cells were pelleted by centrifugation for 10 min at 1500 rpm. The supernatant was carefully removed, and the wash step was repeated twice more using cold culture medium. After the final wash, 1 mL of culture medium was added, and the cells were counted.

### 2.6. Quantitative Real-Time PCR

To obtain RNA from the studied groups of splenocytes, macrophages, and erythroblasts, RNA extraction was performed using TRIzol reagent (Thermo Fisher, 15596026, Waltham, MA, USA), followed by RT-PCR. RNA quality was assessed by electrophoresis and subsequent evaluation using a UV transilluminator (GelDocGo Imaging System, BIO-RAD, Hercules, CA, USA). cDNA was synthesized from the isolated RNA. Reverse transcription was performed using an oligo(dT) primer complementary to the poly-A tail of mRNA using the “RNAscribe RT Reverse Transcriptase” kit (Biolabmix, R04-50, Novosibirsk, Russia). Quantitative real-time PCR was performed using the “BioMaster UDG HS-qPCR Lo-ROX SYBR (2×)” kit (Biolabmix, MHR033-400, Novosibirsk, Russia) on a thermal cycler supporting data normalization using the passive reference dye ROX (CFX96, Bio-Rad, Hercules, CA, USA). Pairs of forward and reverse primers for target genes and a housekeeping gene (HPRT1) were selected (Table 1).

Primer synthesis was carried out by Biolabmix Company (Novosibirsk, Russia). Fluorescence detection of SYBR Green I dye was performed during the elongation step at 72 °C (in the absence of non-specific products). Amplification curve analysis and calculation of relative expression levels (∆∆Ct) were performed using the software supplied with the thermal cycler [20]. Relative gene expression was calculated using the comparative Ct (ΔΔCt) method. For each sample, the Ct value of the target gene was normalized to the Ct value of the housekeeping gene HPRT1 to obtain ΔCt (ΔCt = Ct (receptor gene) − Ct (HPRT1). The ΔΔCt was then calculated as ΔΔCt = ΔCt (sample) − ΔCt (calibrator), where the calibrator was the mean ΔCt of the control group (non-pregnant splenocytes). Relative expression levels were calculated using the formula 2^−ΔΔCt^, with the control group set to 1. The stability of HPRT1 expression across all experimental groups was confirmed prior to analysis.

### 2.7. Determination of Chemokine Concentration by Multiplex Analysis

To assess chemokine production, isolated erythroid cells from spleens and placentas were cultured in complete medium for 48 h at a concentration of 1 million/mL. After culture, cells were pelleted by centrifugation, and the culture supernatant was collected and stored at –80 °C until use. In this study, the Mouse Proinflammatory Chemokine Panel 1 (13-plex) with V-Bottom Plate V02 LegendPlex (Biolegend, 741295, San Diego, CA, USA) was used to investigate chemokine expression levels by murine erythroid cells. Data analysis was performed using flow cytometry; for accurate analysis, at least 300 events per analyte were acquired.

### 2.8. Migration Assay

We used a Transwell migration assay to assess the chemotactic potential of placental NECs [21]. NECs were placed in the lower chamber (semipermeable membrane insert (Tissue Culture Plate Insert, TCS 020024, 01.TC.012.0285, Jet Biofil, Guangzhou, China), where they secreted soluble factors (including chemokines). Fluorescently labelled splenocytes (as a source of immune cells) were placed in the upper chamber, and their migration through the membrane was quantified after 16–18 h. This assay allowed us to determine whether placental NECs can attract immune cells in a paracrine manner, which would have implications for their role in shaping the local immune microenvironment at the maternal-fetal interface. Cells could migrate from one chamber to another through the membrane pores in response to chemoattractants. Into the lower wells, 600 μL of a mixture containing complete medium, erythroid cells (220,000), and blocking antibodies against the chemokines CCL-2 (MCP-1) (Biolegend, San Diego, CA, USA, 505912), CCL-4 (Biolegend, San Diego, CA, USA, 625503), CCL-11 (Biolegend, San Diego, CA, USA, 683203), against the surface antigen PD-L1 (Biolegend, San Diego, CA, USA, 123418) at 5 μg/mL, and the TGF-β inhibitor SB 431542 (S4317-5 MG, Sigma-Aldrich, Zwijndrecht, The Netherlands) at a working concentration of 5 μM were added and incubated for 2 h. Subsequently, pre-labelled CFSE-positive mononuclear cells (350,000 cells in 200 μL) were placed into the upper wells. Erythroid cells without blocking antibodies were used as a control, as was spontaneous migration toward culture medium alone. After 16–18 h, cells from the lower wells were collected to assess the number of migrated cells.

### 2.9. Assessment of the Immunosuppressive Function of Erythroid Cells

To evaluate the suppressive effect, erythroid cells from spleens and placentas were co-cultured with CFSE-pre-stained immunocompetent splenocytes for 72 h in 24-well plates (TPP, Trasadingen, Switzerland, 92024). To assess the dose-dependent effect, different erythroid cell:splenocyte ratios were used: 2:1 (2×), 1:1 (1×), and 0.5:1 (0.5×). The baseline number of splenocytes was 400,000 cells per well. Co-culture was performed in a volume of 600 μL. After culture, cells were additionally stained with monoclonal antibodies against CD3 Alexa Fluor^®^ 700 (Biolegend, San Diego, CA, USA, 100216), and the number of proliferating cells [22] within the CD3-positive T-cell population was assessed using an Attune NXT flow cytometer. Splenocytes cultured alone (without erythroid cells) were used as a control. However, under these conditions, no proliferation was observed, so the data are presented as absolute proliferation values without normalization.

### 2.10. Statistical Analysis

Statistical analysis of the results was performed using the GraphPad Prism 8 software package. Normality of the distributions was tested using the Kolmogorov–Smirnov and Shapiro–Wilk tests. When comparing two groups, Student’s *t*-test was used; for comparisons involving more than two groups, ANOVA with multiple-comparison tests was applied. Parametric tests (ANOVA) were used for normally distributed data (CD45, PD-L1, TGF-β, migration, suppression), whereas non-parametric tests (Kruskal–Wallis) were applied when normality assumptions were not met (ROS data).

## 3. Results

### 3.1. Assessment of Expression of the Immunosuppressive Molecules PD-L1 and TGF-β and of Reactive Oxygen Species (ROS) by Placental Erythroid Cells During the Second and Third Trimesters of Pregnancy

**Figure 1:**
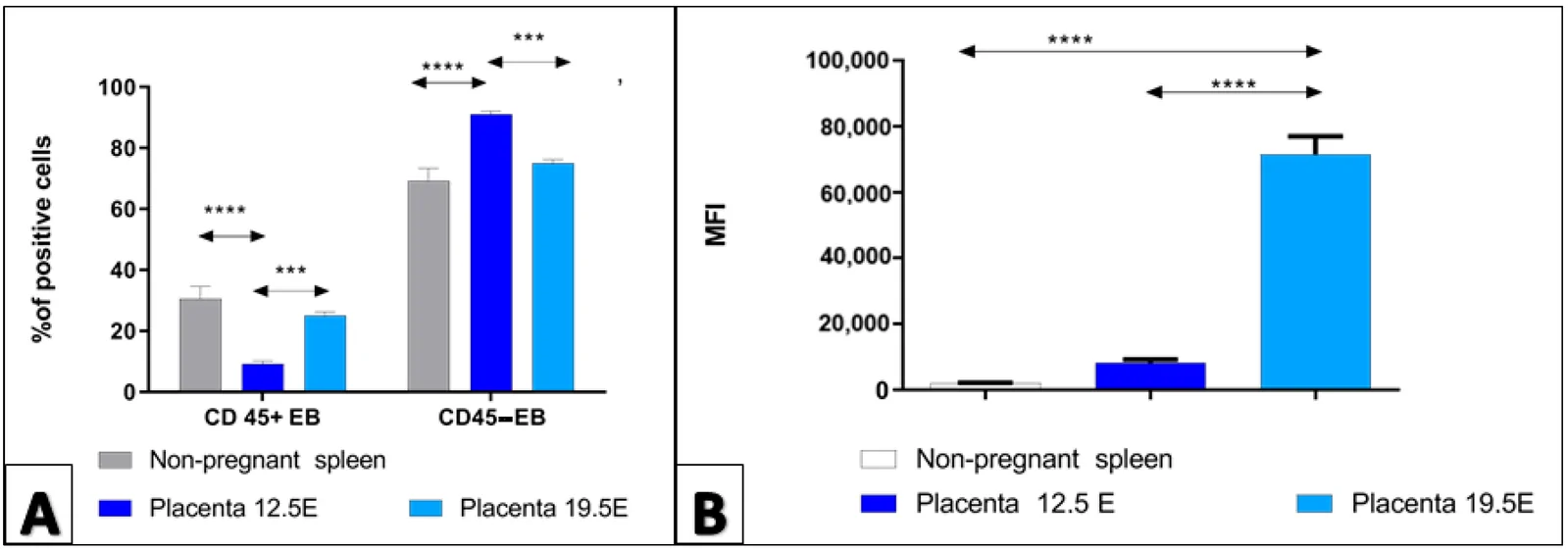
CD45 expression on nucleated erythroid cells from spleen (non-pregnant) and placenta at E12.5 and E19.5. (**A**) Percentage of CD45^+^ cells within the TER-119^+^ population. (**B**) Mean fluorescence intensity (MFI) of CD45. Data are shown as the mean ± SEM (*n* = 6 per group). The TER-119^+^ population comprised >90% of the sorted cells as assessed by flow cytometry. Statistical analysis was performed using one-way ANOVA with Tukey’s post hoc test. *** *p* < 0.001, **** *p* < 0.0001.

Sorted Ter119^+^ spleen cells were used to assess the phenotype of nucleated erythroid cells. The phenotype of nucleated erythroid cells from the placenta was analyzed in the total placental cell population. Figure 1A shows that both CD45-negative and CD45-positive erythroid cells are present in the spleen and placenta of mice during mid and late pregnancy. CD45-positive erythroid cells are considered to possess the most pronounced immunoregulatory potential. Their proportion was minimal at embryonic day 12.5 and corresponded to the level observed in the erythroid cells of the spleen of non-pregnant mice by embryonic day 19.5.

However, analysis of fluorescence intensity, corresponding to the expression level of the surface antigen, showed that splenic erythroblasts are characterized by very low expression levels of the CD45 marker, which increased slightly by mid-gestation and reached a maximum by late gestation (Figure 1B).

**Figure 2:**
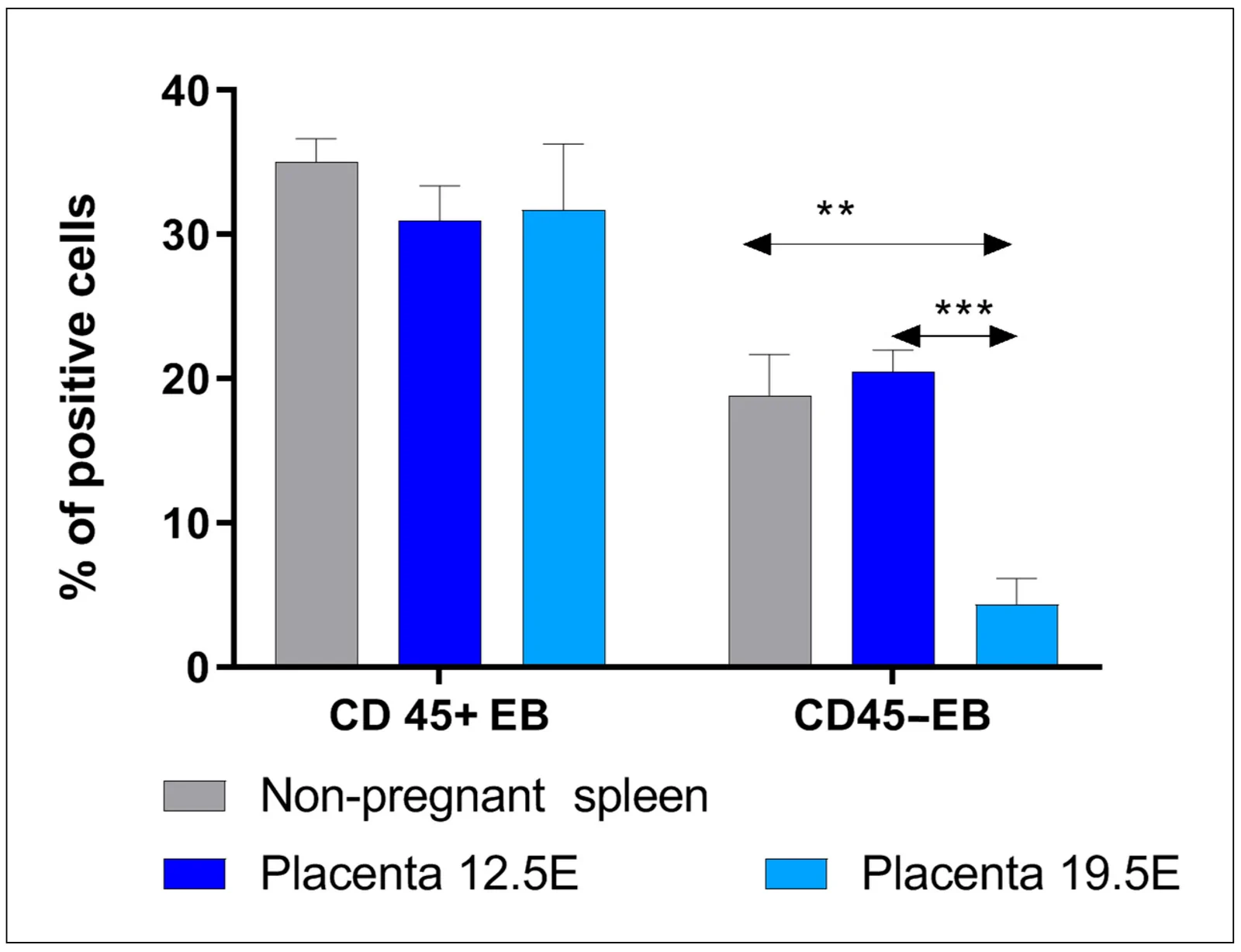
Proportion of PD-L1-positive nucleated erythroid cells in the spleen (non-pregnant) and placenta at E12.5 and E19.5. Data are shown as the mean ± SEM (*n* = 6 per group). Statistical analysis was performed using one-way ANOVA with Tukey’s post hoc test. ** *p* < 0.01, *** *p* < 0.001.

Analysis of PD-L1 expression showed that this molecule is present on both CD45-positive and CD45-negative erythroid cells, with predominance in the CD45-positive subset. In CD45-positive placental erythroid cells, the proportion of PD-L1-positive cells did not change during pregnancy and remained comparable to that in splenic erythroid cells of non-pregnant mice. Among CD45-negative erythroid cells, the proportion of PD-L1-positive cells did not change relative to splenic erythroid cells at embryonic day 12.5, whereas a significant decrease was observed by late gestation (Figure 2).

**Figure 3:**
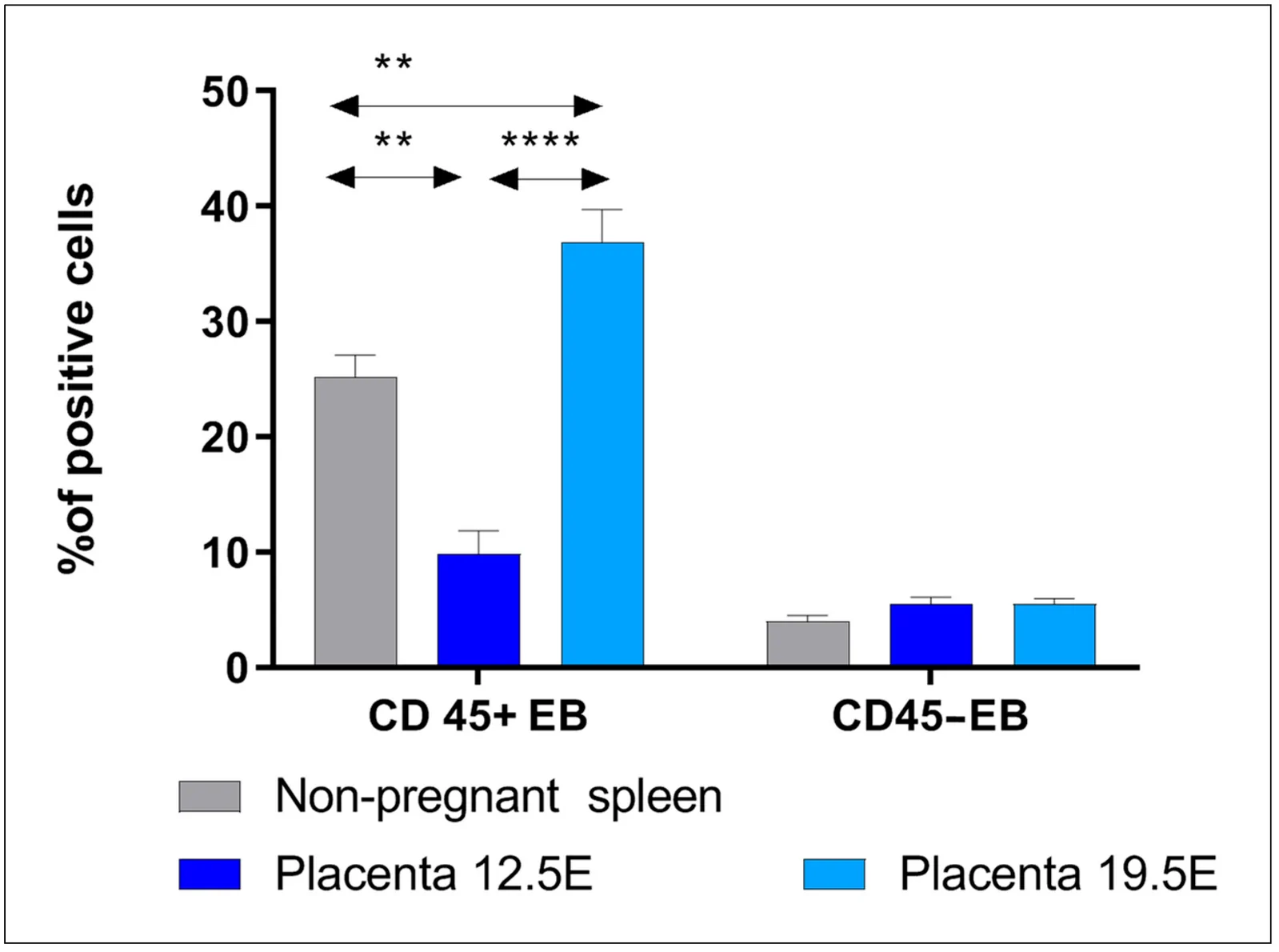
Proportion of TGF-β -positive nucleated erythroid cells in the spleen (non-pregnant) and placenta at E12.5 and E19.5. Data are shown as the mean ± SEM (*n* = 6 per group). Statistical analysis was performed using one-way ANOVA with Tukey’s post hoc test. ** *p* < 0.01, **** *p* < 0.0001.

Analysis of TGF-β-expressing cells among nucleated erythroid cells from the spleen of non-pregnant mice and the placenta of pregnant mice showed that CD45-positive erythroid cells are the main producers of TGF-β, with their peak proportion occurring at late gestation (Figure 3).

For the isolation and phenotyping of erythroid cells, we used antibodies directed against TER-119 from a single clone (BioLegend), although with different conjugates (biotinylated for magnetic sorting and FITC-conjugated for flow cytometry). According to the manufacturer, these antibodies recognize the same TER-119 epitope, which may theoretically introduce a risk of competitive binding when applied sequentially. To minimize this potential interference, we performed thorough washing steps after sorting, employed saturating antibody concentrations for phenotyping, and included isotype control antibodies in all flow cytometric analyses.

Production of reactive oxygen species is one of the prominent immunosuppressive mechanisms employed by nucleated erythroid cells. CD45-positive erythroid cells were shown to be the main producers of ROS. CD45-negative erythroid cells produced much lower amounts of ROS.

**Figure 4:**
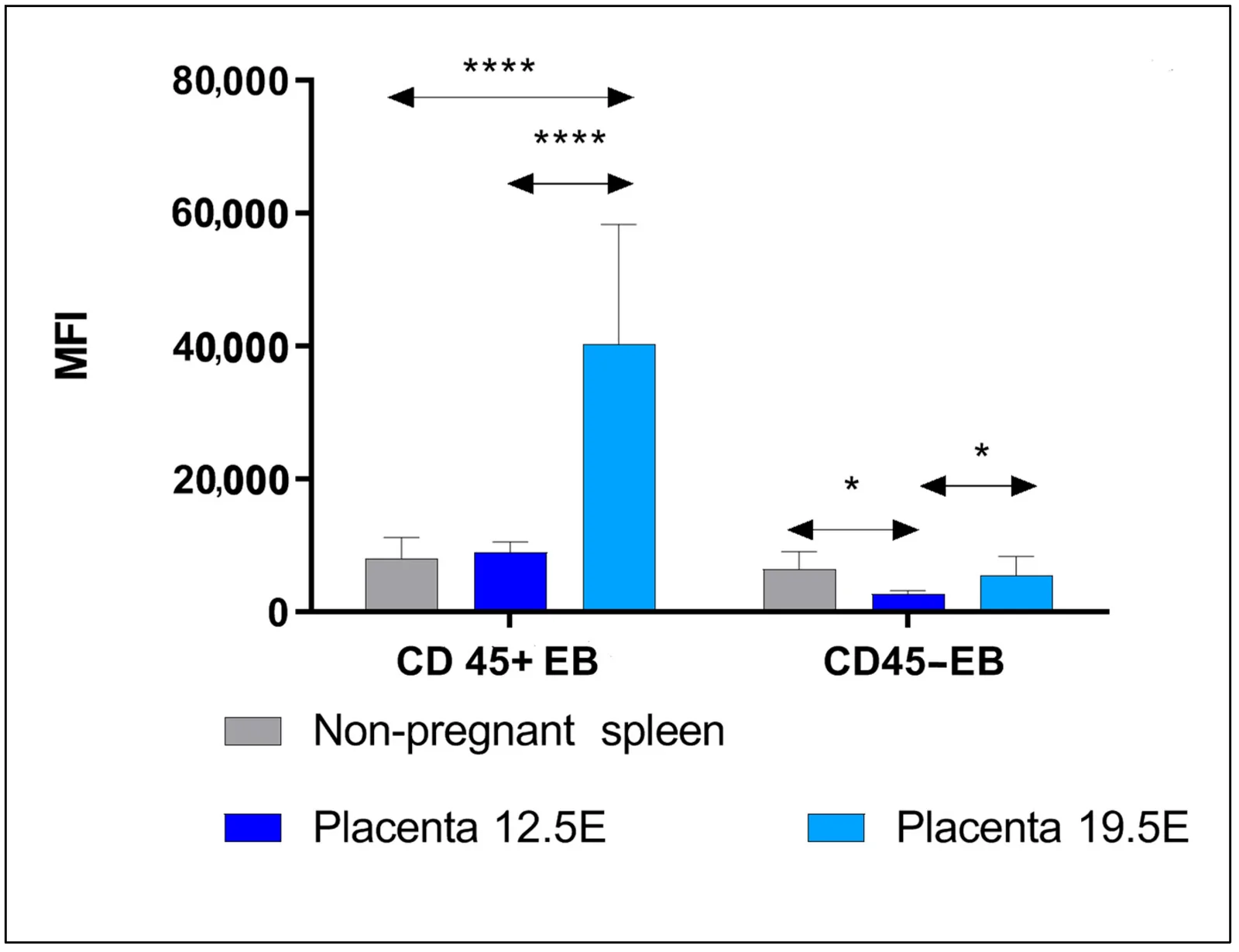
Production of reactive oxygen species (ROS) by nucleated erythroid cells from spleen (non-pregnant) and placenta at E12.5 and E19.5. Mean fluorescence intensity (MFI) of ROS within the TER-119^+^ population. Data are shown as median and interquartile range (*n* = 6 per group). Statistical analysis was performed using the Kruskal–Wallis test with Dunn’s multiple-comparison test. * *p* < 0.05, **** *p* < 0.0001.

Analysis of fluorescence intensity showed that the highest level of ROS production was characteristic of CD45^+^ placental erythroid cells at late gestation (Figure 4).

### 3.2. Determination of the Chemokine Production Profile of Nucleated Erythroid Cells in Each of the Studied Conditions

**Figure 5:**
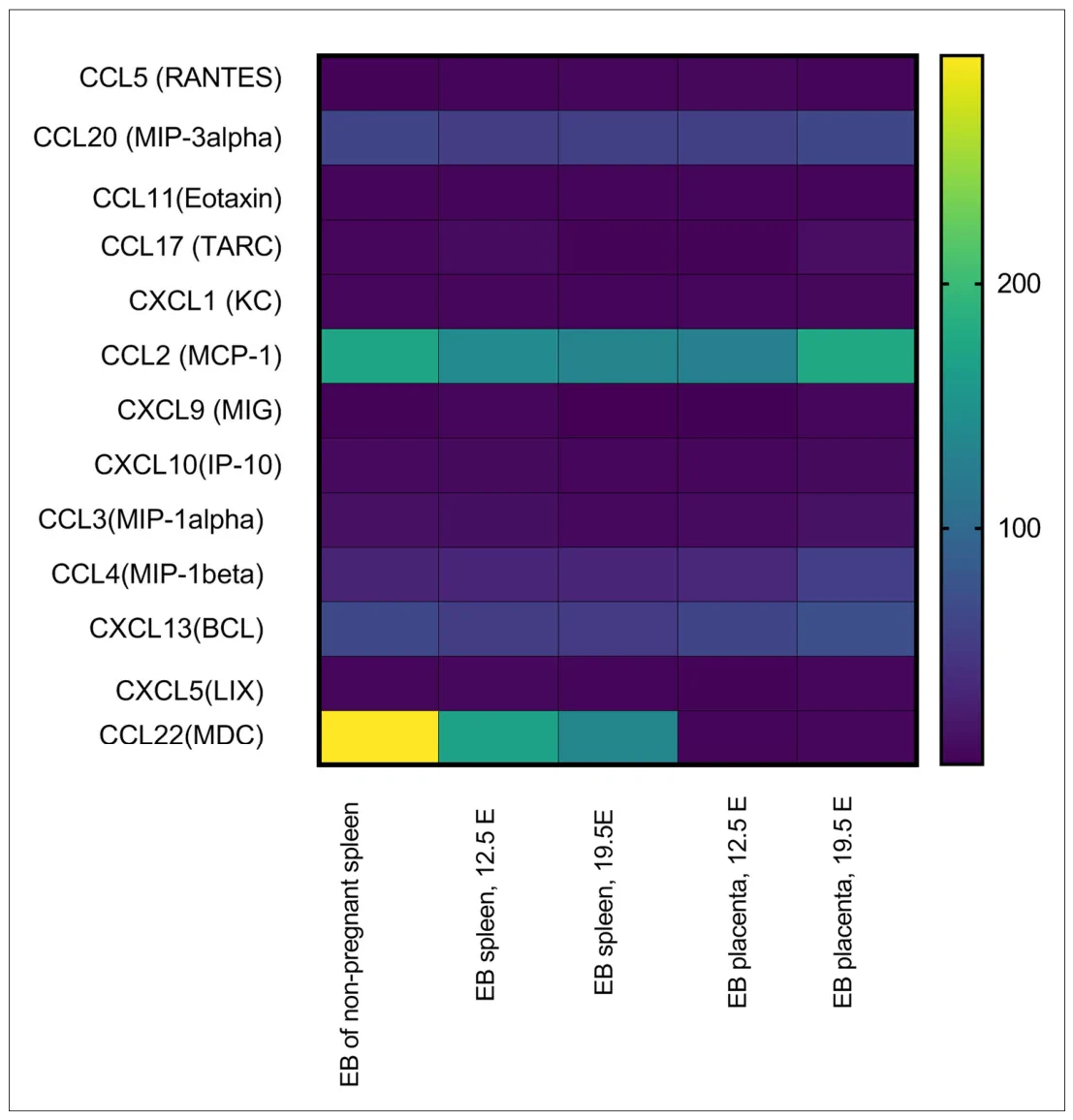
Heat map of chemokine expression by nucleated erythroid cells from the spleens (*n* = 6) of pregnant and intact mice and from the placentas of pregnant mice (*n* = 6 per gestational age).

TER-119-positive cells obtained by positive magnetic sorting were cultured for 48 h after isolation from the spleens of non-pregnant mice and from the spleens and placentas of pregnant mice at embryonic days 12.5 and 19.5. To assess chemokine production levels, a LegendPlex panel (Biolegend) was used, comprising CCL5 (RANTES), CCL20 (MIP-3α), CCL11 (Eotaxin), CCL17 (TARC), CXCL1 (KC), CCL2 (MCP-1), CXCL9 (MIG), CXCL10 (IP-10), CCL3 (MIP-1α), CCL4 (MIP-1β), CXCL13 (BLC), CXCL5 (LIX), and CCL22 (MDC). The panel includes chemokines of both the CC (mainly attracting monocytes, lymphocytes, and eosinophils) and CXC (neutrophils, lymphocytes, angiogenesis) subfamilies and allows assessment of various aspects of the immune response: Th1 inflammation, Th2 inflammation, recruitment of innate immune cells, lymphoid neogenesis, and angiogenesis (Figure 5).

**Figure 6:**
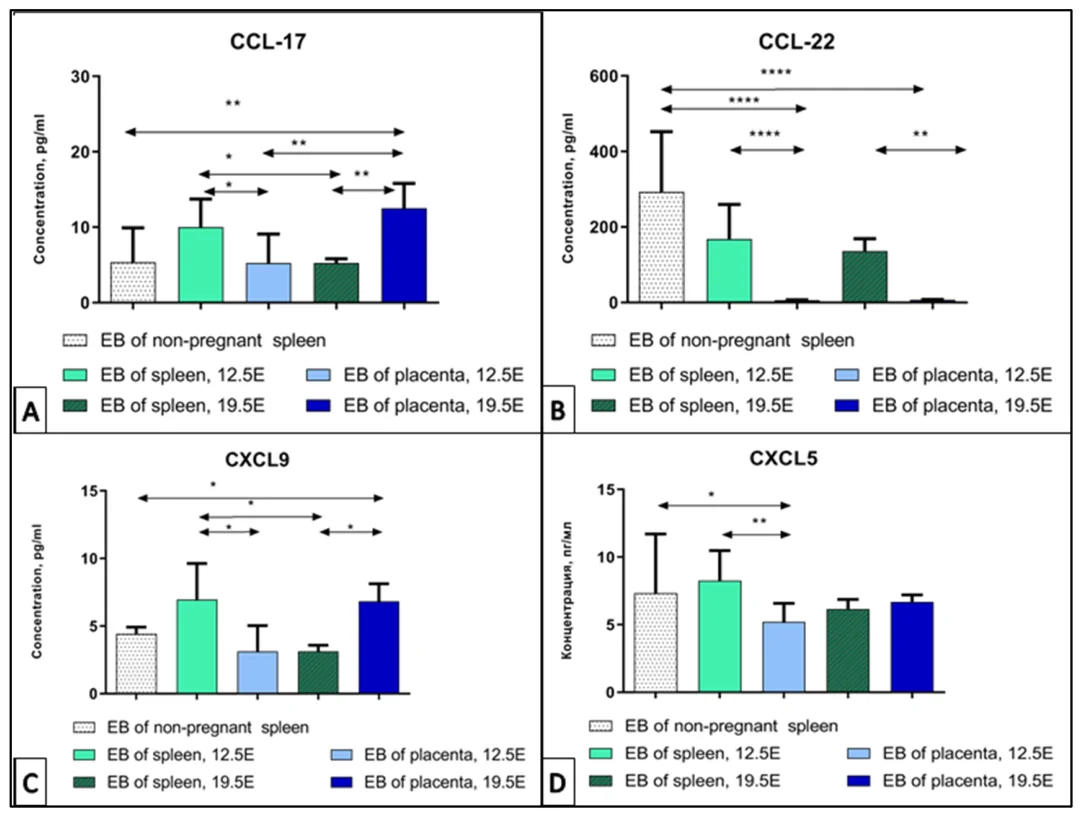
Production of chemokines CCL17 (**A**), CCL22 (**B**), CXCL9 (**C**), and CXCL5 (**D**) by nucleated erythroid cells from spleen (non-pregnant) and placenta and spleen at E12.5 and E19.5. Data are shown as median and interquartile range (*n* = 6 per group). Statistical analysis was performed using the Kruskal–Wallis test with Dunn’s multiple-comparison test. * *p* < 0.05, ** *p* < 0.01, **** *p* < 0.0001.

Erythroid cells were shown to produce all the chemokines studied. Most observed differences were characteristic of the chemokines CCL17, CCL22, CXCL9, and CXCL5 (Figure 6). The highest CCL17 production (Figure 6A) was characteristic of placental erythroid cells at embryonic day 19.5. At E12.5, CCL17 production predominated in splenic rather than placental erythroid cells, whereas by E19.5, CCL17 production had shifted to placental erythroid cells. At mid-gestation, splenic erythroid cells were more active producers of CCL17. By late gestation, CCL17 production by splenic erythroid cells decreased, while CCL17 production by placental erythroid cells significantly increased.

Chemokine CCL22 production (Figure 6B) was maximal for splenic erythroid cells from non-pregnant mice. This same level of chemokine production was maintained in splenic erythroid cells at both mid- and late gestation. Placental erythroid cells produced very small amounts of CCL22, and this production did not change over the course of the studied gestational period (Figure 6B).

CXCL9 9. chemokine production (Figure 6C) increased during pregnancy compared to the non-pregnant state. At E12.5, CXCL9 production predominated in splenic rather than placental erythroid cells, whereas by E19.5, CXCL9 production had shifted to placental erythroid cells. At mid-gestation, splenic erythroid cells were more active producers of CXCL9. By late gestation, CXCL9 production by splenic erythroid cells decreased, while CXCL9 production by placental erythroid cells significantly increased.

CXCL5 chemokine production (Figure 6D) by splenic erythroid cells at E12.5 corresponded to that produced by erythroid cells of non-pregnant mice. CXCL5 production by placental erythroid cells at E19.5 was significantly lower compared to that produced by splenic erythroid cells. This level of CXCL5 chemokine production was maintained until late gestation.

### 3.3. Assessment of mRNA Levels of the Receptors CCR1, CCR2, CCR3, CCR4, CCR5, CXCR1, CXCR2, CXCR3, and CXCR5 in Nucleated Erythroid Cells, Macrophages, and Other Immunocompetent Cells to Evaluate Their Chemotactic Potential

Nucleated erythroid cells are capable of producing a wide range of cytokines and chemokines. A critically important aspect is the sensitivity to chemotactic signals of both the erythroid cells themselves and the cells toward which their immunoregulatory activity may be directed. This sensitivity determines their capacity for directed migration, interaction with other immune cells, integration into sites of inflammation or immune response, and depends directly on the repertoire of chemokine receptors expressed on the cell surface. To address this, we assessed mRNA levels of key chemokine receptors, allowing inference of the cells’ fundamental ability to respond to major classes of chemoattractants. Splenic erythroid cells from non-pregnant mice, immunocompetent splenocytes (TER-119-negative cells), and macrophages derived from bone marrow cells under M-CSF stimulation were used to study chemokine receptor expression. Forward and reverse primer pairs for the target and housekeeping genes were selected.

**Figure 7:**
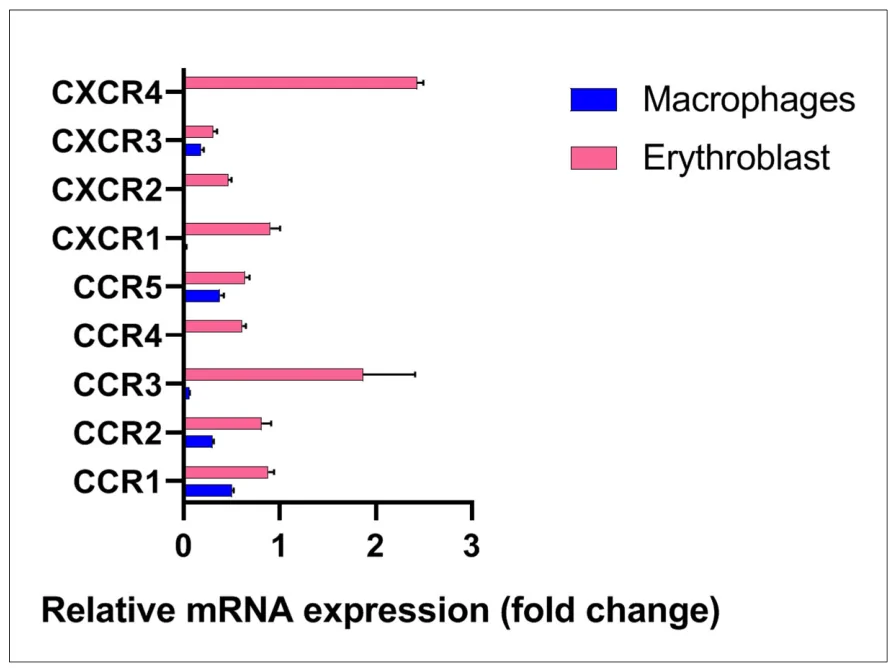
Expression of chemokine receptor genes in macrophages and erythroblasts from non-pregnant mice. Relative mRNA expression was normalized to the housekeeping gene HPRT1 using the ΔΔCt method. Relative expression is shown as fold change (2^(–ΔΔCt)) relative to the non-erythroid splenocytes, set to 1. Data are shown as the mean ± SEM (*n* = 4 per group).

The PCR results were interpreted using the ΔΔCt method (comparative Ct method), which determines the fold change in target gene expression relative to the control group (immunocompetent TER-119-negative splenocytes). The expression levels of the receptors CCR3, CCR4, CXCR1, CXCR2, and CXCR4 in macrophages were much lower than in erythroid and immunocompetent cells. The expression levels of CCR1, CCR5, and CXCR3 were somewhat higher but still much lower than in erythroid and immunocompetent cells. This effect may be explained by the fact that the studied macrophages were derived from earlier bone marrow precursors, had not been stimulated via Toll-like receptors, and, in terms of properties and phenotype, more closely resembled resting tissue macrophages. Accordingly, such macrophages exhibit low sensitivity to chemotactic stimuli. When comparing chemokine receptor expression in nucleated erythroid cells and splenocytes, it was shown that erythroid cells predominantly express CCR3 and CXCR4. CXCR4 expression in erythroid cells was more than twice the level of the housekeeping gene. CCR1, CXCR4, and CCR2 expression levels were comparable in erythroid cells and splenocytes (Figure 7).

The quantitative PCR data unequivocally demonstrate that splenic nucleated erythroid cells possess a rich and active repertoire of chemokine receptors. This directly indicates their fundamental ability to perceive a wide range of chemotactic signals from the microenvironment, which constitutes the molecular basis for their presumed migratory activity and integration into immune processes. Comparative analysis revealed a unique feature of NECs: they exhibit predominant expression of CCR3 and CXCR4 compared to the total splenocyte population. While CCR1, CCR2, and CXCR1 expressions were comparable between erythroid and non-erythroid cell populations, the CCR3 and CXCR4 profiles clearly differentiate erythroid cells. This confirms that erythroid cells represent a specialized population with immunoregulatory potential distinct from that of the general lymphoid cell pool. The extremely low mRNA levels of most of the studied chemokine receptors in cultured macrophages likely reflect their phenotype as resting, non-activated, resident macrophages. This result indicates that in vitro-derived macrophages without additional stimulation possess minimal baseline chemotactic readiness, in contrast to nucleated erythroid cells.

### 3.4. Assessment of Immune Cell Migration Toward Nucleated Erythroid Cells from the Mouse Placenta During the Second and Third Trimesters of Pregnancy

**Figure 8:**
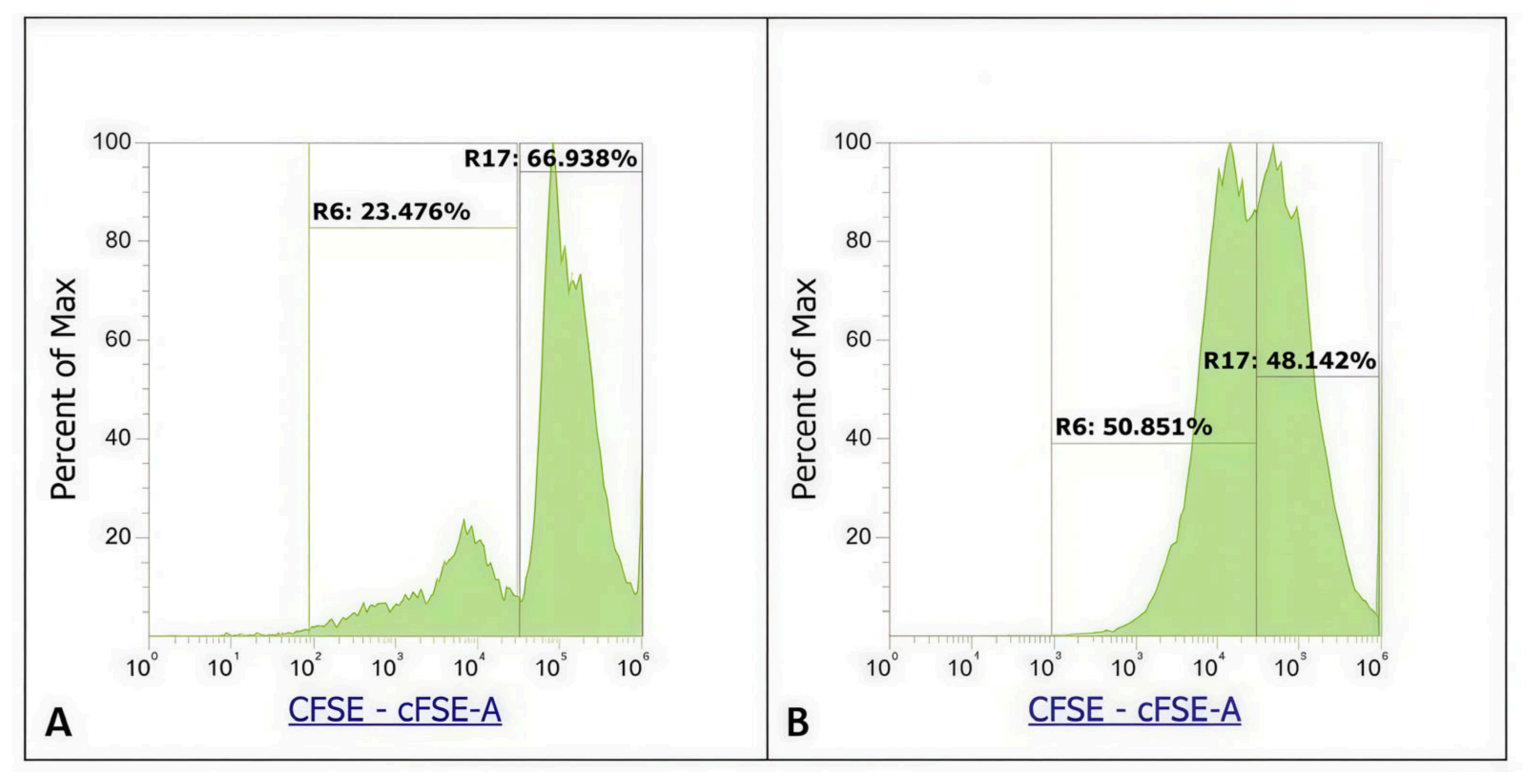
Histograms showing the distribution of the original and proliferating populations of splenic T-cells under the influence of erythroid cells. (**A**) Proliferative activity of splenic T-cells from intact mice in response to erythroid cells from intact mice; (**B**) proliferative activity of splenic T-cells from pregnant mice in response to placental erythroid cells. R17—original population of CFSE-labelled cells; R6—proliferating cells. Representative histograms from a single experiment are shown; quantitative summary of all experiments is presented in Figure 9.

Transwell system plates were used to evaluate the migratory potential of erythroid cells. Erythroid cells, along with blocking antibodies against the chemokines CCL2, CCL4, and CCL11, against the surface antigen PD-L1, and an inhibitor of secreted TGF-β, were placed into the lower wells of the plates. Splenocytes pre-stained with CFSE were placed into the upper wells. After 16–18 h, migrated cells were counted using a flow cytometer. Figure 8 shows that splenic erythroid cells from non-pregnant mice exhibited the greatest capacity to attract immunocompetent cells. Placental erythroid cells at embryonic day 12.5 had a significantly reduced migratory potential, which remained at the same level at embryonic day 19.5. When blocking antibodies against CCL2 and CCL4 were used, a significant increase in the migratory activity of placental erythroid cells at E19.5 was observed compared to E12.5. PD-L1 and TGF-β blockers had no effect on suppressive activity (Table 2).

### 3.5. Assessment of the Immunosuppressive Function of Nucleated Erythroid Cells from the Mouse Placenta During the Second and Third Trimesters of Pregnancy

**Figure 9:**
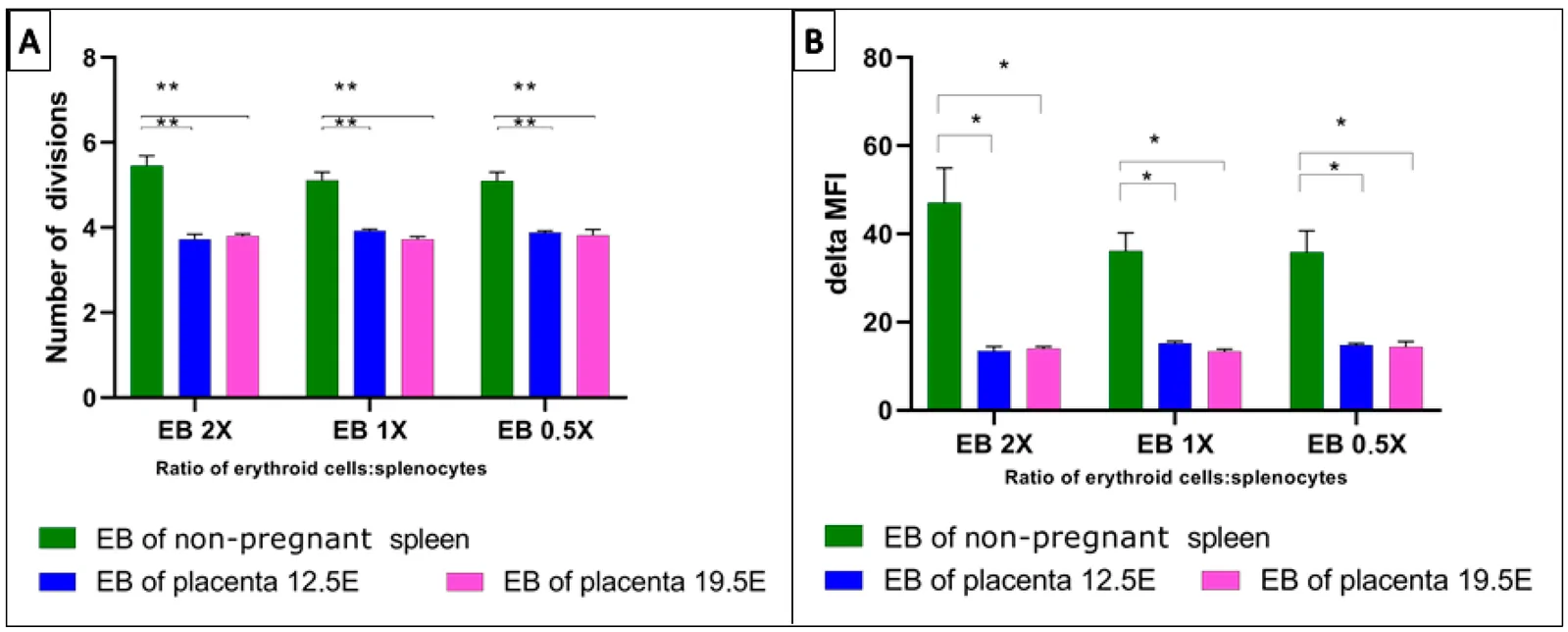
Immunosuppressive activity of nucleated erythroid cells from spleen (non-pregnant) and placenta at E12.5 and E19.5. T-cell proliferation was assessed by CFSE dilution after co-culture with NECs. (**A**) Proliferation level (delta MFI). (**B**) Number of cell divisions. Splenocytes alone (without erythroid cells) were used as a control; no proliferation was detected under these conditions. Data are shown as the mean ± SEM (*n* = 6 per group). Statistical analysis was performed using one-way ANOVA with Tukey’s post hoc test. * *p* < 0.05, ** *p* < 0.01.

The immunosuppressive function of nucleated erythroid cells from the mouse placenta during the second and third trimesters of pregnancy was assessed by their ability to inhibit the proliferation of immunocompetent splenocytes. To this end, splenocytes were stained with CFSE and then incubated with different numbers of erythroid cells: a 2-fold excess (2×), an equal ratio (1×), and a lower number (0.5×). During flow cytometric analysis, the T-cell population was identified, followed by the distribution of the original and proliferating populations. Figure 8 shows representative histograms of the distribution of the original and proliferating T-cell populations. Representative histograms from a single experiment are shown; a quantitative summary of all experiments is presented in Figure 9. It was shown that in non-pregnant mice, a smaller number of cells entered proliferation in response to erythroid cells compared to pregnant mice.

Since the fluorescence intensity of CFSE decreases by half with each cell division, the proliferation intensity was defined as:

delta MFI = (MFI of the original population R17)/(MFI of the proliferating population R6)

Based on the obtained result, the number of divisions undergone by each T-cell population in response to erythroid cells was determined:

number of divisions = log_2_(delta MFI)

Figure 9 shows that for splenocytes from non-pregnant mice in response to splenic erythroid cells, the proliferation intensity was significantly higher than that of splenocytes from pregnant mice in response to placental erythroid cells at all studied erythroid cell: splenocyte ratios. No significant differences in proliferation level were observed between E12.5 and E19.5. When assessing the number of cell divisions, splenocytes from non-pregnant and pregnant mice underwent approximately six and four divisions in response to erythroid cells, respectively.

Functional assays (migration and suppression) were conducted on the total TER-119^+^ population without prior fractionation into CD45^+^ and CD45^−^ subsets. Although we showed that CD45^+^ cells are the predominant producers of PD-L1, TGF-β, and ROS, we cannot definitively conclude that all observed functional effects are mediated exclusively by this subpopulation. It should also be noted that separating CD45^+^ and CD45^−^ erythroid cells presents substantial technical difficulties: two sequential positive magnetic sorting steps would be required, which would compromise the purity of the target population; additionally, erythroid cells display markedly reduced viability upon flow cytometric sorting, and the overall procedure for obtaining sufficient biological material is prolonged and labour intensive.

## 4. Discussion

The traditional view of erythroid cells as passive oxygen carriers has been fundamentally revised over the past decade, as accumulating evidence has revealed their active and versatile immunoregulatory functions. This is supported by a growing body of evidence demonstrating their involvement in autoimmune diseases [23,24], viral infections [25], neoplastic processes [26,27], infectious diseases [28,29], and anemia [30]. During pregnancy, erythroid cells from the spleen and placenta play a critical role in maintaining feto–maternal tolerance [15]. A recent multi-omic study by Perik-Zavodskaia and colleagues [17] revealed that erythroid cells constitute the predominant immunoregulatory population in murine placentas—on average, 80% and 40% of mononuclear cells at E12.5 and E19.5, respectively. These cells express genes involved in MHC-II antigen presentation (Ctss, Cd74, H2-Aa, H2-Ab1), immune checkpoints (PD-L1), chemokines (Ccl2, Ccl3, Ccl4, Ccl9, Cxcl1, Cxcl12), and antimicrobial peptides (S100a8, S100a9). Our study extends these observations by providing the first functional characterization of the chemokine profiles, migratory capacity, and immunosuppressive activity of placental erythroid cells across gestation.

Our data demonstrate that CD45^+^ erythroid cells represent a substantial population of placental cells. The proportion of CD45^+^ NECs was minimal at E12.5 and increased by E19.5 to levels comparable to those in the spleens of non-pregnant mice. CD45^+^ NECs were the predominant producers of PD-L1, TGF-β, and ROS, with maximal expression of all three markers at E19.5.

These findings are consistent with the seminal work of Delyea and colleagues [16], who first demonstrated that CD71^+^ erythroid cells are critical mediators of feto–maternal tolerance. They showed that these cells suppress TNF-α and IFN-γ production through arginase-2 and PD-L1, and that their depletion leads to fetal rejection. Our study extends these observations in three important ways. We demonstrate that the suppressive function may be primarily attributable to the CD45^+^ subset of erythroid cells, as these cells are the main producers of PD-L1, TGF-β, and ROS. However, since functional assays were performed on the total TER-119^+^ population, this attribution remains a hypothesis that requires direct experimental confirmation. We show that placental erythroid cells simultaneously employ three distinct mechanisms—PD-L1, TGF-β, and ROS—rather than relying on a single pathway. We show that the maximal activity of all three mechanisms coincides with the third trimester, suggesting a time-dependent amplification of suppressive capacity.

The combination of contact-dependent suppression (PD-L1), secretory suppression (TGF-β), and metabolic suppression (ROS) creates a redundant and robust system of immune protection that is resilient to the disruption of individual mechanisms [31,32,33,34,35,36,37]. The maximal activation of these mechanisms in the third trimester likely reflects an evolutionary adaptation to counteract the increasing antigenic pressure from the rapidly growing fetus, while simultaneously preventing premature activation of the inflammatory cascade of labour. This interpretation is supported by the observation that the third trimester is characterized by maximal placental blood flow and a high risk of intrauterine infection [38,39,40]. Importantly, Dunsmore and colleagues [15] demonstrated that CD71^+^ cell function—including TGF-β and ROS production—is impaired in pregnant women with inflammatory bowel disease, correlating with adverse pregnancy outcomes [41]. Our data indicate that in normal pregnancy, these mechanisms are fully active, particularly in the third trimester, reinforcing the concept that intact erythroid cell function is essential for successful gestation.

In our previous study, we demonstrated that murine placental erythroid cells are mainly represented by immunosuppressive CD45^+^ cells and secrete CXCL1, CCL2, CCL3, and CCL4 [42]. In this study, we expanded the chemokine panel to 13 chemokines and revealed that placental erythroid cells produce a much broader range of chemoattractants than previously appreciated.

CCL17 and CXCL9 production shifted from the spleen to the placenta as pregnancy progressed. At E12.5, these chemokines were predominantly produced by splenic NECs, whereas by E19.5, the placenta had become the primary source. This organ-specific and time-dependent switch indicates the establishment of local chemokine regulation in the feto–placental zone.

CCL17 (TARC) is a ligand for CCR4 and mediates the migration of Th2 cells, regulatory T cells, and Th17 cells. It is required for CD4^+^ T-cell activation and differentiation, promoting Th2 cell expansion [43]. Our data suggest that, at mid-gestation, CCR4^+^ Treg recruitment occurs remotely from the spleen, which functions as a central reservoir of immunoregulatory erythroid cells. By late gestation, as the fetus grows substantially, the placenta itself begins to produce CCL17, establishing a local gradient for direct Treg recruitment into the feto–placental zone. This interpretation aligns with the study by Rapp and colleagues, who demonstrated that CCL22—a related CCR4 ligand—controls immunity by promoting Treg communication with dendritic cells in lymph nodes [44]. While CCL22 serves a homeostatic function in secondary lymphoid organs, our data suggest that CCL17 is dynamically regulated during pregnancy to support local Treg recruitment at the feto–maternal interface.

A similar dynamic was observed for CXCL9 (MIG). CXCL9 binds to CXCR3 and attracts CXCR3^+^ cells, including cytotoxic T lymphocytes, natural killer cells, and regulatory T cells [45]. CXCL9 production by placental erythroid cells at E19.5 appears paradoxical at first glance, as CXCL9 is traditionally associated with Th1-mediated inflammation. However, this apparent contradiction may be resolved by two complementary observations. First, controlled inflammation is an integral part of implantation, and local moderate Th1 activity is part of normal early pregnancy, consistent with an inflammatory response regulated by Tregs [5,46,47]. Second, the CXCR3 receptor is expressed on a subset of Tregs that possess potent immunoregulatory properties [48]. CXCR3^+^ Tregs have been shown to be highly suppressive and to accumulate at sites of Th1-type inflammation, where they limit excessive tissue damage. Thus, CXCL9 production by the placenta at the end of gestation may be aimed at recruiting these suppressor Tregs rather than pro-inflammatory effectors, thereby maintaining the balance between tolerance and the controlled inflammation required for successful pregnancy.

Our data also revealed a fundamental difference in the regulation of two chemokines that share the same receptor. CCL22 production by placental erythroid cells was minimal at all gestational stages, whereas splenic cells constitutively produced high levels of this chemokine. CCL22 (MDC), like CCL17, is a ligand for CCR4; however, CCL22 belongs to the group of chemokines that are constitutively expressed under homeostatic conditions and induced upon inflammation [44]. In the absence of inflammation, CCR4 is predominantly expressed by regulatory T cells [49], and CCL22 serves as an important factor for inducing Treg migration in vitro and in vivo [50].

Our data demonstrate that these two functionally similar chemokines are regulated in fundamentally different ways in the context of pregnancy. The persistently high CCL22 levels in the spleen likely maintain the basal Treg pool in this organ independently of gestational age. This suggests that even chemokines sharing the same receptor (CCR4) can have fundamentally distinct regulatory mechanisms during pregnancy. While CCL22 may fulfil a homeostatic function by maintaining basal Treg levels in the spleen, CCL17 appears to be inflammation-regulated, with its production switching to the placenta in late gestation to support local Treg populations. This differential regulation underscores the complexity of the chemokine network at the feto–maternal interface.

Placental NECs produced significantly lower amounts of CXCL5 compared with splenic cells, and this level remained unchanged throughout pregnancy. CXCL5 (LIX), the murine analogue of human ENA-78, primarily attracts neutrophils via the CXCR2 receptor [51]. The low CXCL5 levels in the placenta likely reflect the need to limit infiltration by activated neutrophils, which could damage fetal tissues through the release of reactive oxygen species and proteolytic enzymes. This interpretation is consistent with studies demonstrating that excessive neutrophil infiltration in the placenta is associated with preterm labour and chorioamnionitis [52]. In contrast, the more pronounced CXCL5 production in the spleen may be associated with the protective functions of neutrophils against bacterial infections, a role that is critical for systemic immune defence.

Splenic NECs expressed high mRNA levels of CCR3 and CXCR4 compared with non-erythroid splenocytes and macrophages. CXCR4 is the sole receptor for CXCL12 (SDF-1), and the CXCL12/CXCR4 interaction mediates homing of haematopoietic stem cells to the bone marrow [53]. Furthermore, CXCR4 is expressed on erythroid cells [54], and bone-marrow-derived CXCL12 activates CXCR4 on mouse erythroblasts, promoting their differentiation rather than migration through intracellular and nuclear CXCR4 signalling [55]. We hypothesize that during pregnancy, when CXCL12 is produced by stromal cells of the placenta and decidua [56], splenic NECs may be recruited to the placenta to perform suppressive functions. This is consistent with the role of the CXCL12/CXCR4 axis in cell retention and homing in haematopoietic niches.

CCR3 is the main receptor for eotaxins (CCL11, CCL24, CCL26) [57]. Upon eotaxin binding, CCR3 modulates the microenvironment towards a Th2-dominant state [58]. The predominance of CCR3 and CXCR4 on NECs suggests that these cells are simultaneously capable of retention in splenic stromal niches (via CXCR4) and rapid migration in response to Th2-associated inflammatory signals (via CCR3). Unlike most lymphocytes, which use CCR7 to enter lymph nodes [59], NECs may utilize alternative migratory signals to exit the spleen and home to sites of Th2-type inflammation, such as the placenta. In contrast, macrophages derived from bone marrow upon M-CSF stimulation exhibited extremely low mRNA levels for most of the chemokine receptors examined. This does not imply that macrophages do not express these receptors at all; rather, it reflects their resting, unactivated phenotype, since most functional macrophage receptors are induced upon stimulation (e.g., by LPS or IFN-γ). This observation is consistent with the classical view of macrophage polarization, where receptor expression is dynamically regulated by environmental cues.

Our data indicate that placental NECs (both at E12.5 and E19.5) possess significantly lower migratory activity in attracting splenocytes compared with splenic NECs from non-pregnant mice. Despite producing a broad spectrum of chemokines, the reduced migratory activity of placental NECs may be explained by the presence of not only chemoattractants but also chemorepellents, which were not assessed in this study. Alternatively, immunocompetent cells during pregnancy circulate between the maternal and fetal compartments and may already be desensitized to certain chemokines. This reduced migratory capacity likely represents an adaptive strategy to limit excessive immune cell infiltration into the placenta, thereby preserving the immunosuppressive niche required for fetal survival.

Since we previously demonstrated high production of CCL2, CCL3, CCL4, and CXCL1 by placental erythroid cells [42], we investigated the effect of blocking CCL2 and CCL4 on chemotactic activity. Unexpectedly, CCL2 and CCL4 blockade enhanced splenocyte migration towards placental NECs at E19.5, contrary to the expected reduction.

A plausible explanation for this unexpected observation involves atypical chemokine decoy receptors, particularly ACKR2 (D6). ACKR2 is expressed on trophoblasts and binds a wide range of inflammatory CC chemokines, including CCL2, CCL4, and CCL17, but does not activate G-protein-dependent chemotactic signalling [60,61]. By blocking CCL2 and CCL4, we may have disrupted the competitive equilibrium for ACKR2 binding, thereby liberating other chemoattractants such as CCL17 and CXCL9, which are produced by placental erythroid cells at E19.5. This hypothesis is supported by the observation that the paradoxical effect was observed only at E19.5, when CCL17 and CXCL9 production by placental erythroid cells peaks. Alternative explanations, including antibody cross-reactivity or off-target effects, cannot be completely excluded but are unlikely given the validated specificity of the blocking antibodies used. Future studies using ACKR2-deficient mice or specific ACKR2 inhibitors will be required to definitively test this hypothesis.

In co-culture experiments, NECs from the placenta markedly suppressed T-cell proliferation. T-cells exposed to placental NECs underwent only approximately four divisions, compared with six divisions when exposed to splenic NECs from non-pregnant mice. Full suppressive function of placental NECs was established by mid-gestation (E12.5) and maintained until full term. This is consistent with the high risk of fetal rejection in the first half of pregnancy; protective mechanisms must be activated as early as possible and operate continuously.

Notably, despite the observed remodelling of the chemokine profile between E12.5 and E19.5, suppressive activity remained unchanged. This suggests that NECs can produce different chemokine signals depending on pregnancy needs while maintaining consistently high suppressive function. Consistent with this interpretation, PD-L1 and TGF-β blockade did not affect migratory function. This independence of chemotactic and suppressive functions is an important finding, as it indicates that these two properties are regulated through distinct pathways.

Several methodological limitations should be acknowledged. First, we did not distinguish between fetal and maternal erythroid cells in the placenta. In the murine placenta, both fetal and maternal erythroid cells are present, and their relative proportions may change with gestational age. Our TER-119^+^ population from the placenta likely represents a mixed population.

Second, antibodies to TER-119 from a single clone (BioLegend) were used for both isolation and phenotyping, albeit with different conjugates. According to the manufacturer, these antibodies recognize the same epitope, which may introduce a risk of competitive binding. To minimize this, we performed extensive washing and used saturating antibody concentrations, but we acknowledge that using antibodies against different epitopes would be methodologically preferable.

Third, functional assays were performed on the total TER-119^+^ population without prior separation into CD45^+^ and CD45^−^ subpopulations. Although we demonstrated that CD45^+^ cells are the main producers of PD-L1, TGF-β, and ROS, we cannot definitively attribute all observed functional effects to this subset. Separating erythroid cells into CD45^+^ and CD45^−^ fractions presents considerable technical challenges, including reduced viability and cell loss during sorting. Future studies using partial depletion of CD45^+^ cells or FACS sorting of CD45^+^ and CD45^−^ fractions will be required to directly test this hypothesis. Fourth, our functional assays were performed in vitro, which may not fully recapitulate the complex tissue architecture and multicellular interactions present in vivo. For example, Transwell migration assays assess only soluble factors and do not account for the effects of extracellular matrix or cell–cell contacts.

**Figure 10:**
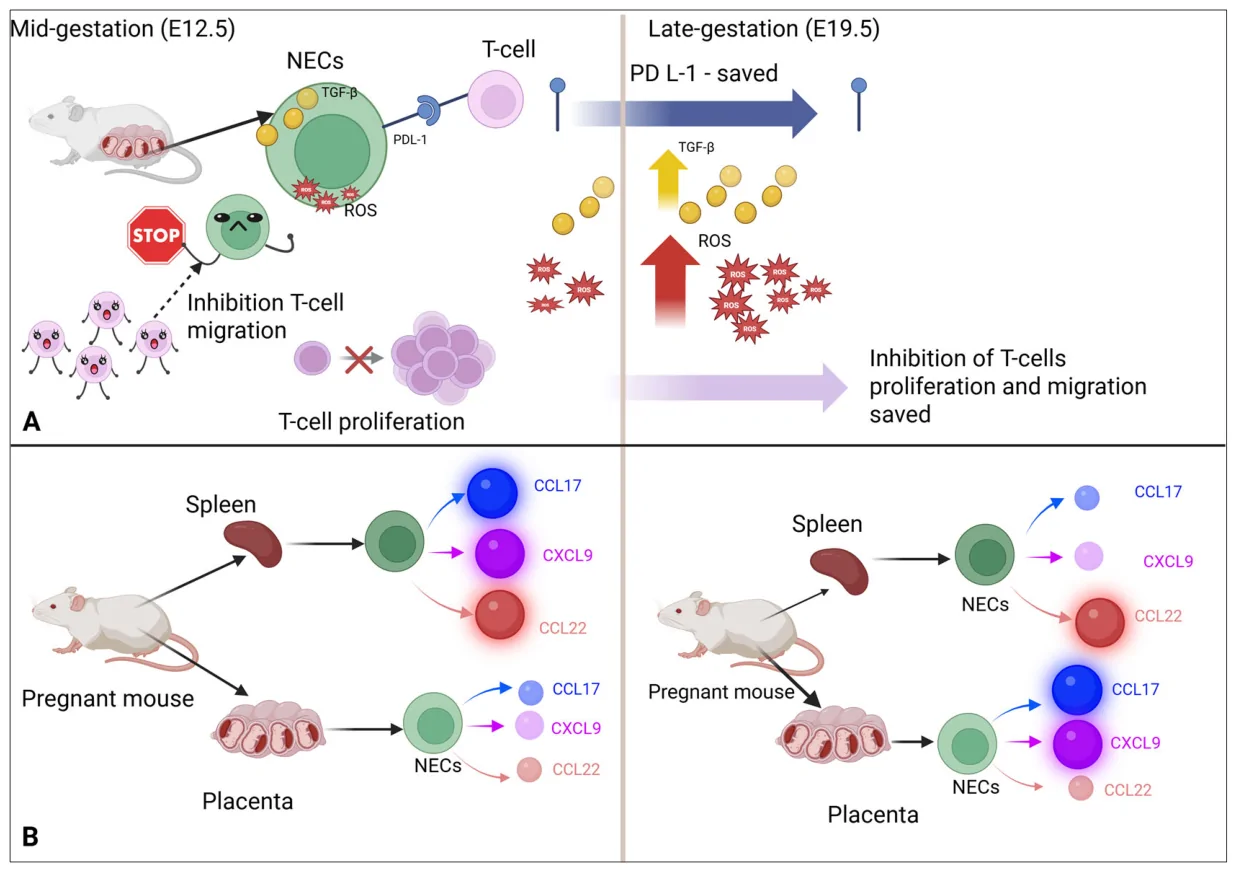
Schematic summary of the main findings. (**A**) Immunosuppressive activity and T-cell regulation. At mid-gestation (E12.5), placental nucleated erythroid cells (NECs) already exhibit immunosuppressive activity through three mechanisms: PD-L1 (contact-dependent suppression), TGF-β (secreted, promoting Treg induction), and ROS (metabolic suppression). At late gestation (E19.5), the activity of all three mechanisms is maintained or enhanced. Placental NECs suppress T-cell proliferation. The suppressive capacity remains stable from E12.5 to E19.5. Placental NECs exhibit reduced migratory activity towards immunocompetent cells compared with splenic NECs. (**B**) Chemokine profiles. At mid-gestation (E12.5), splenic NECs produce high CCL17 and CXCL9 levels. CCL22 is constitutively produced at high levels in the spleen. As pregnancy progresses (E12.5 → E19.5), CCL17 and CXCL9 production shifts from the spleen to the placenta. In contrast, CCL22 remains high in the spleen and minimal in the placenta, suggesting a homeostatic role.

Despite these limitations, the consistent trends observed across multiple independent experiments, together with appropriate controls, support the validity of our conclusions. The key findings of this study are summarized schematically in Figure 10.

## 5. Conclusions

In this study, we provide a comprehensive characterization of the chemokine profiles, receptor repertoire, and immunosuppressive properties of murine placental nucleated erythroid cells (NECs) during pregnancy.

CD45^+^ placental NECs are a major source of PD-L1, TGF-β, and ROS, with maximal expression at E19.5, forming a multimodal immunosuppressive system at the maternal–fetal interface. CCL17 and CXCL9 production shifts from the spleen at mid-gestation to the placenta at late gestation (E12.5 → E19.5), indicating the establishment of local chemokine regulation in the feto–placental zone. In contrast, CCL22 is constitutively high in the spleen and minimal in the placenta, suggesting a homeostatic role. Splenic NECs express high CCR3 and CXCR4 levels, suggesting a dual functional capacity: retention in the stromal niche and readiness for migration in response to Th2-associated signals. Paradoxical enhancement of migration upon CCL2/CCL4 blockade at E19.5 suggests the involvement of atypical chemokine decoy receptors, though this remains preliminary and requires further validation. Despite dynamic chemokine remodelling, the immunosuppressive capacity of placental NECs remains stable from mid-gestation onwards, indicating independent regulation of chemotactic and suppressive functions.

These findings contribute to our understanding of the immunobiology of pregnancy and may have implications for complications such as preterm labour and preeclampsia. Future studies exploring the molecular mechanisms underlying the paradoxical chemokine effect and the functional differences between CD45^+^ and CD45^−^ erythroid subsets will further elucidate the role of these cells in maintaining successful pregnancy.

## Figures and Tables

**Table 1:** Pairs of forward and reverse primers.

Gene	Forward Sequence (5′ → 3′)	Reverse Sequence (5′ → 3′)
CCR1	GCCAAAAGACTGCTGTAAGAGCC	GCTTTGAAGCCTCCTATGCTGC
CCR2	GCTGTGTTTGCCTCTCTACCAG	CAAGTAGAGGCAGGATCAGGCT
CCR3	CCACTGTACTCCCTGGTGTTCA	GGACAGTGAAGAGAAAGAGCAGG
CCR4	GGACTAGGTCTGTGCAAGATCG	TGCCTTCAAGGAGAATACCGCG
CCR5	GTCTACTTTCTCTTCTGGACTCC	CCAAGAGTCTCTGTTGCCTGCA
CXCR1	CCATTCCGTTCTGGTACAGTCTG	GTAGCAGACCAGCATAGTGAGC
CXCR2	CTCTATTCTGCCAGATGCTGTCC	ACAAGGCTCAGCAGAGTCACCA
CXCR3	TACGATCAGCGCCTCAATGCCA	AGCAGGAAACCAGCCACTAGCT
CXCR5	ATCGTCCATGCTGTTCACGCCT	CAACCTTGGCAAAGAGGAGTTCC
HPRT1	CTGGTGAAAAGGACCTCTCGAAG	CCAGTTTCACTAATGACACAAACG

**Table 2:** Migratory activity of nucleated erythroid cells from spleen (non-pregnant) and placenta at E12.5 and E19.5. Splenocyte migration towards NECs was assessed using Transwell assays. Migrated cells are shown as a percentage of the initial immunocompetent cell population.

	EB of Non-Pregnant Spleen	EB of Placenta, 12.5E	EB of Placenta, 19.5E
EB + medium	62.73 ±4.61	28.53 ± 3.55 **	41.64 ± 2.16 *
EB + CCL2	66.48 ± 4.55	25.45 ± 2.07 ****	43.60 ± 1.78 **, #
EB + CCL4	61.01 ± 4.73	20.51 ± 1.05 ****	43.03 ± 4.52 *, ##
EB + CCL11	57.89 ± 4.91	32.76 ± 4.41 *	45.80 ± 6.46 *
EB + PD-L1	61.76 ± 8.17	32.09 ± 0.91 ***	44.20 ± 5.03 *
EB + TGF	57.52 ± 6.18	32.04 ± 2.51 ***	42.85 ± 4.01 *

Data are shown as the mean ± SEM (*n* = 6 per group). Statistical analysis was performed using one-way ANOVA with Tukey’s post hoc test. * *p* < 0.05, ** *p* < 0.01, *** *p* < 0.001, **** *p* < 0.0001 compared to the group “EB of non-pregnant spleen”; # *p* < 0.05, ## *p* < 0.01, compared to the group “EB of placenta, 12.5E”.

## Data Availability

The original contributions presented in this study are included in the article. Further inquiries can be directed to the corresponding author.

## Abbreviations

The following abbreviations are used in this manuscript:

ANOVA	Analysis of variance
CCL	C-C motif chemokine ligand
CCR	C-C chemokine receptor
CD	Cluster of differentiation
CFSE	Carboxyfluorescein succinimidyl ester
CXCL	C-X-C motif chemokine ligand
CXCR	C-X-C chemokine receptor
E12.5	Embryonic day 12.5 (mid-gestation)
E19.5	Embryonic day 19.5 (late gestation)
FBS	Fetal bovine serum
HEPES	4-(2-hydroxyethyl)-1-piperazineethanesulfonic acid
HPRT1	Hypoxanthine phosphoribosyltransferase 1
mRNA	Messenger ribonucleic acid
NEC	Nucleated erythroid cell
NK cell	Natural killer cell
PBS	Phosphate-buffered saline
PCR	Polymerase chain reaction
qPCR	Quantitative real-time polymerase chain reaction
ROS	Reactive oxygen species
RT-PCR	Reverse transcription polymerase chain reaction
TER-119	Erythroid lineage marker (mouse)
UV	Ultraviolet
